# Lack of association of the *CIITA *-168A→G promoter SNP with myasthenia gravis and its role in autoimmunity

**DOI:** 10.1186/1471-2350-11-147

**Published:** 2010-10-13

**Authors:** Ryan Ramanujam, Yaofeng Zhao, Ritva Pirskanen, Lennart Hammarström

**Affiliations:** 1 Division of Clinical Immunology, Department of Laboratory Medicine, Karolinska Institutet at Karolinska University Hospital Huddinge, SE-141 86 Stockholm, Sweden; 2 Department of Neurology, Karolinska Hospital Solna, SE-171 76 Stockholm, Sweden

## Abstract

**Background:**

The major histocompatibility complex class II transactivator (CIITA) regulates MHC class II gene expression. A promoter SNP -168A→G (rs3087456) has previously been shown to be associated with susceptibility to several immune mediated disorders, including rheumatoid arthritis (RA), multiple sclerosis (MS) and myocardial infarction (MI). Myasthenia gravis (MG) is an autoimmune disorder which has previously been shown to be associated with polymorphisms of several autoimmune predisposing genes, including *IL-1*, *PTPN22*, *TNF-α *and the *MHC*. In order to determine if allelic variants of rs3087456 increase predisposition to MG, we analyzed this SNP in our Swedish cohort of 446 MG patients and 1866 controls.

**Results:**

No significant association of the SNP with MG was detected, neither in the patient group as a whole, nor in any clinical subgroup. The vast majority of previous replication studies have also not found an association of the SNP with autoimmune disorders.

**Conclusions:**

We thus conclude that previous findings with regard to the role of the *CIITA *-168A→G SNP in autoimmunity may have to be reconsidered.

## Background

Myasthenia gravis (MG) is an antibody mediated autoimmune disorder characterized by auto-antibodies against the nicotinic acetylcholine receptor situated on the muscle end-plate. These auto-antibodies impair the transmission of nerve impulses to the muscle. MG patients commonly display thymic abnormalities such as hyperplasia and thymoma and the latter is usually associated with severe disease. MG occurs in 14.1 per 100,000 persons in Sweden, and has a concordance rate of 30-40% in monozygotic twins and 2-3% in dizygotic twins, indicating a strong genetic component. Subgroups of patients have commonly been made based on age of onset, thymic status, and disease severity. Several autoimmune predisposing genes have previously been shown to be associated with MG, including *IL-1*, *PTPN22*, and genes in the major histocompatibility complex (MHC), particularly the human leukocyte antigen (HLA)-B8, DR3 haplotype and *TNF-α *[1].

The class II transactivator (CIITA, GenBank accession number NM_000246), located on chromosome 16p13, is a transactivator of the MHC class II genes [2]. Four alternative promoters, which exhibit cell-type-specific activity, drive transcription of the *CIITA *gene [3]. Expression of MHC class II proteins is crucial for cell collaboration and induction of immune responses, and lack of expression is associated with the severe immunodeficiency disease bare lymphocyte syndrome (BLS) [2].

In view of its suggested role in autoimmune disorders [4], we sought to determine if the *CIITA *rs3087456 variant is associated with autoimmune MG, using a large cohort of Swedish patients.

## Methods

### Patients and controls

This study included 466 unrelated Swedish MG patients and 1866 healthy control individuals of self-reported European ancestry. MG was diagnosed as described previously [5], and clinical information was documented by the primary physician. The controls were derived from blood donors in the Stockholm area (n = 533; adults) and from a population based Swedish material (n = 1333; newborns) [6]. Ethical permission was obtained from the Karolinska Institutet for use of patient and control samples.

### MHC2TA genotyping

Genotyping of the 446 MG cases and 1866 control samples was performed using matrix-assisted laser desorption/ionization time-of-flight (MALDI-TOF) [7] mass spectrometry (SEQUENOM Inc., San Diego, California, USA) at the Mutation Analysis Facility of the Karolinska Institutet, Sweden. PCR was conducted using forward primer ACGTTGGATGCTTCACCAAATTCAGTCCAC and reverse primer ACGTTGGATGTTTACCACACTCCCTTAAGC. The MHC SNP rs3087456 was genotyped using iPLEX chemistry utilizing unextended primer (UEP) CACTCCCTTAAGCCCTCCC and extension primers CACTCCCTTAAGCCCTCCCC and CACTCCCTTAAGCCCTCCCT.

### Statistical analysis

The χ^2 ^test was used to compare genotypes and allele frequencies of the *CIITA *SNP in patients and controls. For the overall MG cohort, a *p*-value below 0.05 was considered to indicate statistical significance. For subsequent analyses, a Bonferroni correction was applied based on the number of subgroups to determine the significance threshold. Power for the study was calculated using "CaTS - Power Calculator for Two Stage Association Studies" (http://www.sph.umich.edu/csg/abecasis/CaTS/) [8]. The study had 80% power to detect allelic odds ratios greater than 1.28 at the stated significance level (α = 0.05), with a MAF of 0.266 using an additive model and 1.39 using a dominant model.

### Patient subgrouping

Due to the complex nature of MG, we stratified the patient material into subgroups based on clinical information to investigate association to potential subclasses of the disease. Patients were thus separated on the basis of thymic histopathology identified postoperatively after thymectomy (not operated, normal, hyperplasia or thymoma), disease severity (ocular, generalized or severe) and by age of onset. A Bonferroni correction for the eight subgroups created was applied to the significance threshold (*p *< 6.25 × 10^-3^) used in subsequent comparisons.

There is a lack of consensus regarding the age which most accurately separates early onset MG (EOMG) from late onset MG (LOMG), with several publications using 50 years of age [9,10] and others using 40 years of age [11]. Therefore, in order to avoid erroneous results, patients with an age of disease onset less than 40 years constituted the EOMG group, while those with age of onset of 50 years or older were included in the LOMG group.

## Results

There were no significant differences in the rs3087456 allele frequency between the blood donor (n = 533; adults) and population based (n = 1333; newborns) control subgroups (*p *= 0.192). Therefore, they were pooled into one group comprising 1866 samples.

The genotyping results for the 446 MG patients and 1866 controls and *p*-values of association of the SNP with MG are given in Table 1. No statistically significant difference was observed between MG patients and controls for either allele frequencies (*p *= 0.092) or genotypes (*p *= 0.251). The strongest associations were in the ocular and EOMG subgroups, with uncorrected p-values of 0.040 and 0.010, respectively. Nevertheless, they yielded non-significant *p*-values when compared with the corrected significance threshold of 6.25 × 10^-3^.

**Table 1:** Results of *CIITA *genotyping in MG patients and controls.

MG patients	AA	AG	GG	A	G	MAF	**p-val**^**a**^	Significance threshold	OR (95% C.I.)	**p-val reference**^**b**^
All patients (n = 446)	260	159	27	679	213	0.239	*0.092*	*0.05*	0.86 (0.73-1.02)	*0.154*
Age of onset <40 (n = 226)	127	80	19	334	118	0.261	*0.810*	*6.25 × 10*^*-3*^	1.03 (0.82-1.28)	*0.032*
Age of onset >50 (n = 175)	110	59	6	279	71	0.203	*0.010*	*6.25 × 10*^*-3*^	1.43 (1.09-1.87)	*0.559*
Ocular (n = 42)	29	12	1	70	14	0.167	*0.040*	*6.25 × 10*^*-3*^	0.55 (0.31-0.98)	*0.274*
Generalized (n = 404)	231	147	26	609	199	0.246	*0.240*	*6.25 × 10*^*-3*^	0.90 (0.76-1.07)	*0.068*
Not operated (n = 171)	103	58	10	264	78	0.228	*0.124*	*6.25 × 10*^*-3*^	0.81 (0.63-1.06)	*0.619*
Normal thymus (n = 60)	35	25	0	95	25	0.208	*0.156*	*6.25 × 10*^*-3*^	0.72 (0.46-1.13)	*0.833*
Thymic hyperplasia (n = 157)	87	59	11	233	81	0.258	*0.747*	*6.25 × 10*^*-3*^	0.96 (0.74-1.25)	*0.090*
Thymoma (n = 58)	35	17	6	87	29	0.250	*0.695*	*6.25 × 10*^*-3*^	0.92 (0.60-1.41)	*0.389*
^a^Controls (n = 1866)	1015	708	143	2738	994	0.266				
^b^Controls (n = 1599)	989	528	82	2506	692	0.216				

Genotypes, frequencies of A and G alleles as well as the minor allele frequency (MAF) for MG patients and various subgroups of patients compared to the measured controls^a ^and the controls from Swanberg et. al.^b ^[4]. The significance threshold applies a Bonferroni correction for eight tests on subsequent subgroup classifications.

## Discussion

Previously, evidence for association between the *CIITA *type III promoter (-168A/G, rs3087456) and rheumatoid arthritis (RA) and multiple sclerosis (MS) was reported [4]. This polymorphism is associated with reduced transcription levels of *CIITA ex vivo *in human leukocytes stimulated with interferon-γ, and *in vivo *experiments in rats have demonstrated a strong correlation between lowered levels of *CIITA *transcripts and reduced expression of MHC class II molecules [4]. We therefore tested our Swedish MG patients and controls for association with this SNP, revealing that no significant difference in the allelic frequencies of the SNP exists between patients and controls and among patients when stratified by clinical subgroups of the disease.

Our Swedish control material differed from the Swedish control material presented by Swanberg et. al. [4] with a significantly higher frequency of the minor (G) allele (0.266 and 0.216, respectively, *p *< 10^-5^). In order to exclude association of MG with rs3087456, given the large variation in control allele frequencies, we also compared MG allele frequencies to this control material. However, the MG group did not show a statistically significant association, nor did any subgroup of MG (Table 1).

Although the initial study on rs3087456 in autoimmune/inflammatory disorders showed a clear association with the minor (G) allele, subsequent studies on a variety of similar disorders have been inconsistent and for the most part have shown a lack of association. Of 17 replication studies using patients from 26 different disease cohorts (Table 2), only four have found an association between rs3087456 and a disorder. In the case of RA, eight replication studies have been conducted in 10 different populations, of which only two [12,13] found an association with rs3087456. In one of the latter, Iikuni et. al. found that although the allele frequencies in controls were similar to those in Sweden, the major (A) allele was associated with an increased risk of RA in a Japanese patient cohort (*p *= 0.003). This result runs contrary to the increased risk of the minor (G) allele reported in a Swedish population [4] and brings the proposed genetic mechanism associated with the minor allele into question. A meta-analysis of the 10 RA data sets consisting of 6861 patients and 9270 controls also determined that neither the G allele (*p *= 0.70) nor the GG genotype (*p *= 0.16) are associated to the disease [14].

**Table 2:** Summary of published *CIITA *SNP rs3087456 association studies in autoimmune disorders.

Study	Year	Population	Disease	Patients/Controls	Association
Swanberg, et. al. [4]	2005	Sweden	**RA**	1262/2506	*p *= 0.012
Swanberg, et. al. [4]	2005	Sweden	MS	520/2506	*p *= 0.028
Swanberg, et. al. [4]	2005	Sweden	MI	376/2506	*p *= 0.014
Koizuma et. al. [20]	2005	Japan	SLE	100/100	Not significant
Yazdani-Biuki et. al. [21]	2006	Austria	**RA**	362/1709	Not significant
Orozco et. al. [12]	2006	Spain	**RA**	748/676	*p *= 0.01^a^
Orozco et. al. [12]	2006	Sweden	**RA**	278/478	Not significant
Orozco et. al. [12]	2006	Argentina	**RA**	287/287	Not significant
Akkad et. al. [22]	2006	Germany	**RA**	319/463	Not significant
Akkad et. al. [22]	2006	Germany	MS	646/463	Not significant
Akkad et. al. [22]	2006	Germany	WG^b^	178/463	Not significant
Eyre et. al. [23]	2006	UK	**RA**	1401/2475	Not significant
Lindholm et. al. [24]	2006	Finland/Sweden	MI	1222/2345	Not significant
Ghaderi et. al. [16]	2006	Italy	AAD^c^	128/406	*p *= 0.003
O'Doherty et. al. [25]	2007	Ireland	**RA**	293/316	Not significant
O'Doherty et. al. [25]	2007	Ireland	MS	440/316	Not significant
O'Doherty et. al. [25]	2007	Ireland	JIA^d^	74/316	Not significant
Linga-Reddy et. al. [17]	2007	Sweden	SLE	334/478	Not significant
Martinez et. al. [26]	2007	Spain	**RA**	350/519	Not significant
Martinez et. al. [26]	2007	Spain	MS	396/519	Not significant
Martinez et. al. [26]	2007	Spain	IBS^e^	663/519	Not significant
Iikuni et. al. [13]	2007	Japan	**RA**	1121/450	*p *= 0.003^f^
Harrison et. al. [27]	2007	UK	**RA**	733/613	Not significant
Sánchez et. al. [28]	2008	SLE	SLE	394/514	Not significant
Pan-Hammarström, et. al. [29]	2008	Sweden	CVID^g^	97/1826	Not significant
Pan-Hammarström, et. al. [29]	2008	Sweden	IgAD^h^	249/1826	Not significant
Skinningsrud, et. al. [15]	2008	Norway	AAD^c^	332/1029	*p *= 0.044^i^
Dema et. al. [30]	2009	Spain	CD^j^	607/794	Not significant
Bronson, et. al. [19]	2010	USA/UK	MS	1320/1363	Not significant

^a^Considered by the authors to be a possible Type I error

^b^Wegener's granulomatosis

^c^Autoimmune Addison's disease

^d^Juvenile idiopathic arthritis

^e^Inflammatory bowel syndrome

^f^Association with the major (A) rather than the minor allele of rs3087456

^g^Common variable immunodeficiency

^h^IgA deficiency

^i^Uncorrected p-value in a study of 139 SNPs.

^j^Celiac disease

In the 16 data sets from studies which have examined the association of the SNP with other autoimmune/inflammatory disorders than RA, only two have reported a positive association to the SNP, both with autoimmune Addison's disease [15,16]. In the first study, the *p*-value (*p *= 0.044) was not corrected for multiple testing in a study of 139 SNPs, drawing the association into question. One replication study in patients with myocardial infarction as well as four in multiple sclerosis in different populations did not find any association with rs3087456. Furthermore, a Swedish study, which found no association of the SNP with SLE, had a MAF of 0.253, in 956 controls, similar to that in our controls (*p *= 0.41), and the authors concluded that the differences in allele frequencies between their controls and those of Swanberg et. al. could not be explained by population substructure [17].

Although there is evidence of potential interaction between SNPs in *CIITA *[18] and HLA alleles [19] in autoimmunity, the promoter SNP rs3087456 alone appears to predispose neither to myasthenia gravis nor to autoimmunity in general. A further investigation of interacting factors contributing to altered MHC class II expression in autoimmune disorders is warranted.

## Conclusions

The lack of allelic association of the *CIITA *SNP -168A→G (rs3087456) with MG casts further doubt on the presumed role of this particular SNP in autoimmune disorders. In the case of single gene association, results should be replicated, even in the same population, to confirm their validity. This study also illustrates the importance of large, homogeneous sample sizes, particularly for controls, in order to avoid publication bias.

## Competing interests

The authors declare that they have no competing interests.

## Authors' contributions

RR analyzed data, performed the statistical analysis, interpreted the results and drafted the manuscript. YZ prepared the samples and analyzed data. RP acquired patient material and compiled clinical data. LH conceived of the experiments, interpreted the results and drafted the manuscript. All authors read and approved the manuscript.

## Pre-publication history

The pre-publication history for this paper can be accessed here:

http://www.biomedcentral.com/1471-2350/11/147/prepub
